# DNA Methylation Predicts the Response of Triple-Negative Breast Cancers to All-Trans Retinoic Acid

**DOI:** 10.3390/cancers10110397

**Published:** 2018-10-24

**Authors:** Krysta Mila Coyle, Cheryl A. Dean, Margaret Lois Thomas, Dejan Vidovic, Carman A. Giacomantonio, Lucy Helyer, Paola Marcato

**Affiliations:** 1 Departments of Pathology, Dalhousie University, Halifax, NS B3H 4R2, Canada; krysta.coyle@dal.ca (K.M.C.); deanc@dal.ca (C.A.D.); meg.thomas@dal.ca (M.L.T.); dejan.vidovic@dal.ca (D.V.); carman.giacomantonio@dal.ca (C.A.G.); 2 Departments of Surgery, Dalhousie University, Halifax, NS B3H 4R2, Canada; lhelyer@dal.ca; 3 Departments of Microbiology & Immunology, Dalhousie University, Halifax, NS B3H 4R2, Canada

**Keywords:** retinoic acid, triple-negative breast cancer, DNA methylation, biomarkers

## Abstract

All-trans retinoic acid (atRA) regulates gene expression and is used to treat acute promyelocytic leukemia. Attempts to use atRA in breast cancer without a stratification strategy have resulted in limited overall effectiveness. To identify biomarkers for the treatment of triple-negative breast cancer (TNBC) with atRA, we characterized the effects of atRA on the tumor growth of 13 TNBC cell lines. This resulted in a range of effects that was not predictable based on previously hypothesized predictors of response, such as the levels of atRA nuclear shuttling proteins fatty acid binding protein 5 (FABP5) and cellular retinoic acid binding protein 2 (CRABP2). Transcriptional profiling revealed that atRA induced distinct gene expression changes in the sensitive versus resistant cell lines that were mostly independent of the presence of retinoic acid response elements (RAREs) or peroxisome proliferator response elements (PPREs). Given the importance of DNA methylation in regulating gene expression, we hypothesized that differential DNA methylation could predict the response of TNBCs to atRA. We identified over 1400 sites that were differentially methylated between atRA resistant and sensitive cell lines. These CpG sites predicted the response of four TNBC patient-derived xenografts to atRA, and we utilized these xenografts to refine the profile and identified that as many as 17% of TNBC patients could benefit from atRA treatment. These data illustrate that differential methylation of specific CpGs may be useful biomarkers for predicting the response of patient tumors to atRA treatment.

## 1. Introduction

While breast cancer is commonly represented as one disease, it is highly heterogeneous. Many studies have observed molecular heterogeneity, which is associated with distinct drug responses and clinical outcomes [1,2,3,4,5,6]. Triple-negative breast cancer (TNBC) is a diagnosis of exclusion, encompassing all breast cancers which lack expression of the estrogen receptor (ER) and progesterone receptor (PR), and lack amplification of the human epidermal growth factor receptor 2 (HER2). Thus, TNBC is itself a heterogeneous designation, and the molecular subtyping of TNBC can further identify distinct subtypes including luminal, basal-like, claudin-low or mesenchymal, and HER2-like [4,5,7,8,9]. These classification approaches all demonstrate that basal-like and TNBCs have worse prognoses. Furthermore, the lack of targeted therapies for TNBCs drives research to find improved therapeutics and biomarkers of response to existing therapies to improve outcomes for this subtype [10,11].

All-trans retinoic acid (atRA) is clinically used in the treatment of acute promyelocytic leukemia (APL) and high-risk neuroblastoma [12,13,14], and has potential as a therapeutic option for breast cancer [15,16,17,18]. In APL, atRA acts primarily as a differentiation agent, modulating the effects of the characteristic genetic event (PML-RARa fusion) in APL [19]. In other cancers, atRA is hypothesized to induce apoptosis [19] or modulate an immune response to tumor cells [20]. However, clinical studies with atRA in the treatment of breast cancer have demonstrated little to no success [21,22,23], and recent preclinical work has demonstrated a high degree of variability in the response of breast cancers to atRA treatment [16,24,25,26,27,28,29,30,31]. A review of past studies broadly characterizes TNBC as retinoid-resistant based on in vitro evaluation (Supplementary Table S1). A thorough study characterizing the effects of atRA on TNBC in vivo has not yet been performed and may reveal effects that are not predictable based on predominantly in vitro approaches.

Retinoid-based therapies have low systemic toxicity when compared to conventional chemotherapeutics, and the side-effects of atRA in treatment of APL are predominantly attributed to the differentiation of promyelocytes [32]. This positions atRA as an under-utilized clinical agent which may be of benefit to patients with TNBC and other cancers if profiled accurately for response. The present work characterizes the in vivo response of a panel of TNBC cell line xenografts to systemic atRA treatment and identifies sensitive, resistant, and tumor-promoted responses. We have previously demonstrated that some of the heterogeneity in the transcriptional responses of MDA-MB-231 and MDA-MB-468 cells to atRA can be attributed to variations in DNA methylation [33], and in this study, further investigate the contributions of DNA methylation to atRA sensitivity. We utilize the responses to atRA as determined in this work to supervise the determination of differential gene expression and DNA methylation in atRA-sensitive cell lines and demonstrate a broad predictive profile for atRA sensitivity.

We validate our predictive DNA methylation profile in predicting the response of four TNBC patient-derived xenografts (PDXs) to systemic atRA treatment. By adding the PDXs to the model, we further refine the CpG profiles used to determine sensitivity and demonstrate a potential impact on as many as 17% of TNBC patients. This provides a paradigm for the utilization of differential DNA methylation as a predictive indicator of drug response. Our work demonstrates that atRA may be a clinically relevant choice for patients with TNBC and supports further consideration of atRA as a therapeutic option for TNBC.

## 2. Results

### 2.1. Breast Cancer Cell Lines Display A Wide Range of In Vivo Responses to atRA

**Figure 1:**
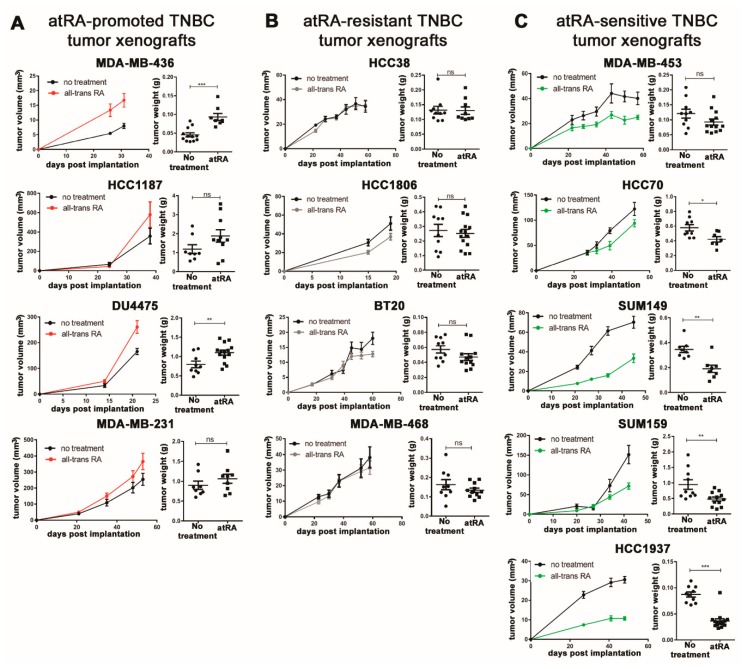
Triple-negative breast cancer (TNBC) cell line xenografts display varied responses to all-trans retinoic acid (atRA) treatment. Treatment of TNBC cell line xenografts in NOD-*scid* mice with 5 mg/60 day slow-release atRA pellets identified distinct phenotypes of (**A**) atRA-promoted, (**B**) atRA-resistant, and (**C**) atRA-sensitive. Tumor weights were compared by student’s *t*-test, * *p* < 0.05, ** *p* < 0.01, *** *p* < 0.001.

**Figure 2:**
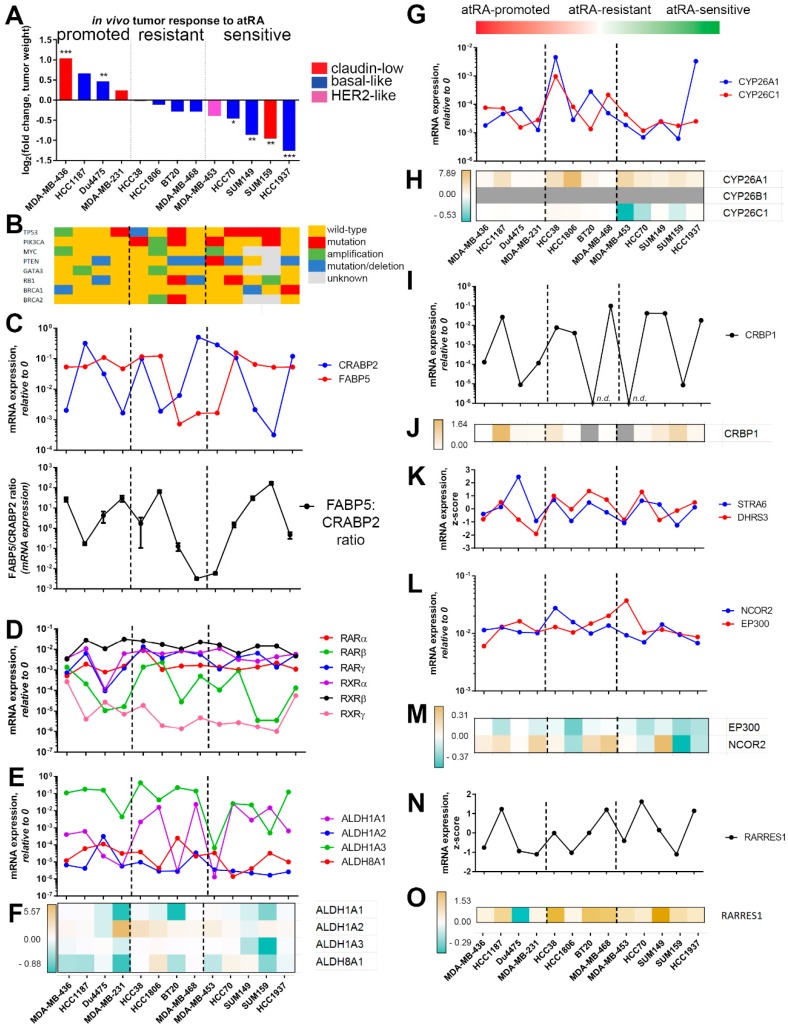
Classical retinoid signaling components do not correlate with atRA sensitivity in TNBC. (**A**) Cell line xenografts are summarized from Figure 1 based on their response to atRA treatment. Values represent fold-change in tumor weight, compared by student’s t-test (* *p* < 0.05, ** *p* < 0.01, *** *p* < 0.001). (**B**) Characterization of known mutations in cell lines from existing databases; dashed line represents distinction for Fisher’s exact test. (**C**) mRNA expression of FABP5 and CRABP2 was determined by qPCR and the ratio plotted. mRNA expression of (**D**) RARα, β, γ, and RXRα, β, γ, (**E**) ALDH1A1, ALDH1A2, ALDH1A3, and ALDH8A1 was determined by qPCR. (**F**) Changes in expression of ALDH isoforms after atRA treatment are represented by color gradient. mRNA expression of (**G**) CYP26A1 and CYP26C1 was determined by qPCR. (**H**) Changes in expression of CYP26 isoforms after atRA treatment are represented by color gradient. (**I**) mRNA expression of CRBP1 and (**J**) changes in expression of CRBP1 after atRA treatment. mRNA expression of (**K**) STRA6 and DHRS3, (**L**) NCOR2 and EP300 are plotted. (**M**) Changes in expression of EP300 and NCOR2 after atRA treatment are represented by color gradient. (**N**) Baseline expression of RARRES1 and (**O**) expression of RARRES1 after atRA treatment.

We investigated the in vivo response of 13 TNBC cell lines to atRA treatment in NOD-*scid* mice and observed a range of effects on tumor volumes and weights (Figure 1). Based on the fold-change of final tumor weight, we characterized four as atRA-promoted (MDA-MB-231, Du4475, HCC1187, and MDA-MB-436), four as atRA-resistant (MDA-MB-468, BT20, HCC1806, and HCC38), and five as atRA-sensitive (HCC1937, SUM159, SUM149, HCC70, and MDA-MB-453). These experiments are summarized as single data points based on tumor weight (Figure 2A).

We identified no significant relationship between molecular subtype and sensitivity to atRA (see Figure 2A). Subsequently, we compared the mutational profiles of these cell lines using data from the Cancer Cell Line Encyclopedia (CCLE) and other sources [34,35,36] to determine if there were any obvious mutations which were correlated with atRA sensitivity. We found no correlation between mutations in breast cancer driver genes TP53, PIK3CA, MYC, PTEN, GATA3, RB1, BRCA1, or BRCA2, and sensitivity to atRA (Figure 2B).

### 2.2. Expression of Retinoid Pathway Genes Does Not Correlate with atRA Sensitivity

We next considered the hypothesis that the expression of retinoid processing and signaling genes dictates the response of breast cancers to atRA treatment. A primary hypothesis for differential effects of atRA is shuttling of atRA by FABP5 in the absence of CRABP2 to peroxisome proliferative response elements (PPREs) in the genome, as opposed to retinoic acid response elements (RAREs) [37,38]. In contrast, we found no correlation between the absolute expression of FABP5 and CRABP2 or the FABP5–CRABP2 ratio and atRA sensitivity (Figure 2C). This is consistent with our previously published data demonstrating limited presence of PPREs in atRA-responsive genes [16,33].

Expression and methylation of RARβ2 [24] is another hypothesized determinant of atRA sensitivity; therefore, we queried both mRNA expression and DNA methylation. We found no relationship between the expression of any RAR or RXR isoforms and atRA sensitivity in the TNBC cell lines profiled (Figure 2D). Additionally, there was no significant correlation between RARβ methylation and atRA sensitivity (Supplementary Figure S1). This does not exclude the possibility that RAR/RXR expression or the FABP5–CRABP2 ratio contribute to divergent gene expression [24,25,39,40]; rather, it suggests that there are limited functional consequences to this hypothesis within the TNBC models used in this study.

Consistent with our previous data [16,41], we confirmed that ALDH1A3 is the most predominant retinaldehyde dehydrogenase mRNA expressed in TNBC (Figure 2E), with the exceptions of HCC70 and SUM159. While the expression of retinaldehyde dehydrogenases may contribute to stem-like characteristics in breast cancer [42,43,44], we identify no correlation between atRA sensitivity and the expression of ALDH1A1, ALDH1A2, ALDH1A3, or ALDH8A1 (Figure 2E); or with differential expression of these isoforms following atRA treatment (Figure 2F).

For a more comprehensive evaluation, we similarly profiled the expression of other retinoid processing and signaling genes (CYP26A1, CYP26B1, CYP26C1, Figure 2G,H; CRBP1, Figure 2I,J; DHRS3, STRA6, Figure 2K; and NCOR2, EP300, Figure 2L,M) and identified no significant correlations with atRA sensitivity. While we previously characterized the role of an atRA-inducible tumor suppressor, RARRES1, in TNBC [41], basal expression of RARRES1 (Figure 2N) and fold-change in expression following atRA treatment (Figure 2O) show no correlation with atRA sensitivity. These findings demonstrate that atRA sensitivity in TNBC is dependent on other factors, which are likely secondary to the classical retinoid signaling pathway.

### 2.3. Differential Gene Expression Is Identified in atRA-Sensitive Cell Lines

**Figure 3:**
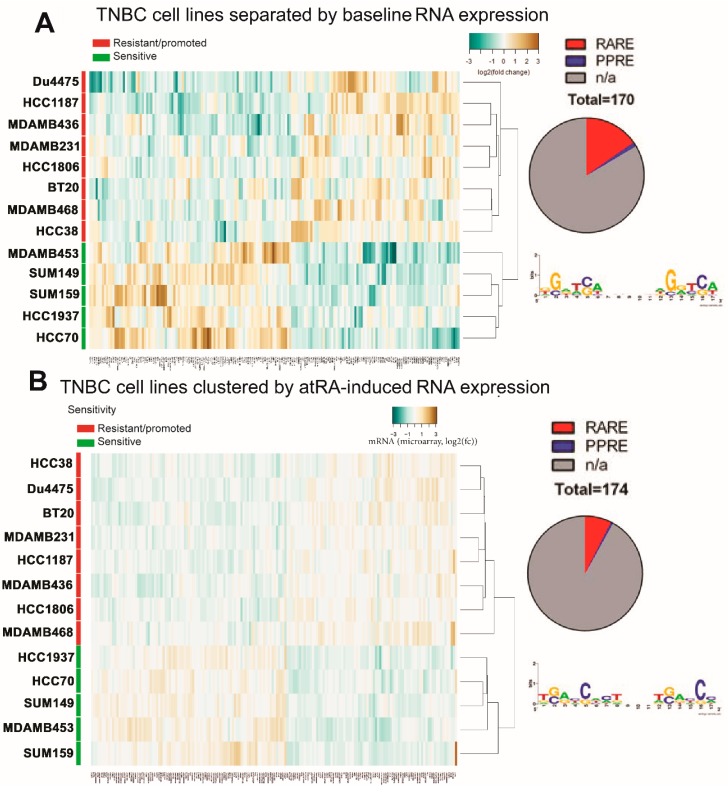
Gene expression varies between TNBC cell lines. (**A**) Differential baseline gene expression and (**B**) differential atRA-induced gene expression were determined between atRA-sensitive cell lines and all other cell lines and hierarchically clustered. Corresponding number of genes neighboring RAREs and PPREs are displayed.

To determine the unknown factors affecting atRA sensitivity in TNBC, we performed gene expression analyses using Affymetrix Human Gene 2.0ST arrays in triplicate (GSE103426 and GSE117579). When atRA-sensitive cell lines were compared to all other TNBC cell lines profiles, we identified 170 transcripts which were differentially expressed at basal levels (Figure 3A, Supplementary File 1); 26 transcripts were within 10 kB of a RARE sequence. We similarly identified 174 transcripts which were differentially expressed following atRA treatment (Figure 3B, Supplementary File 2). Only 13 transcripts were near a RARE sequence, suggesting that differential atRA responses were predominantly RARE-independent and likely due to secondary responses. Ankyrin-repeat domain 6 (ANKRD6) was identified by both data sets. This suggests that while the induced genes could be informative in deciphering the mechanisms of atRA-induced tumor growth versus regression, they have little predictive value alone in the context of untreated patients with breast cancer. On the other hand, differential baseline gene expression may have predictive value.

Furthermore, considering that few canonical atRA-inducible genes were identified in this comparative analysis (sensitive versus other cell lines), we confirm that the microarray analysis identified numerous classical RA target genes in analyses performed on individual cell lines (Supplementary File 3). Cut-offs (absolute log2 fold-change greater than 0.7, *p*-value < 0.01) demonstrate that many of the classic RA target genes such as DHRS3, CYP26A1, and STRA6, although not induced in every cell line, are induced in many of the cell lines.

### 2.4. DNA Methylation Contributes to Differential Gene Expression between atRA-Responsive and -Resistant TNBC Cell Lines

**Figure 4:**
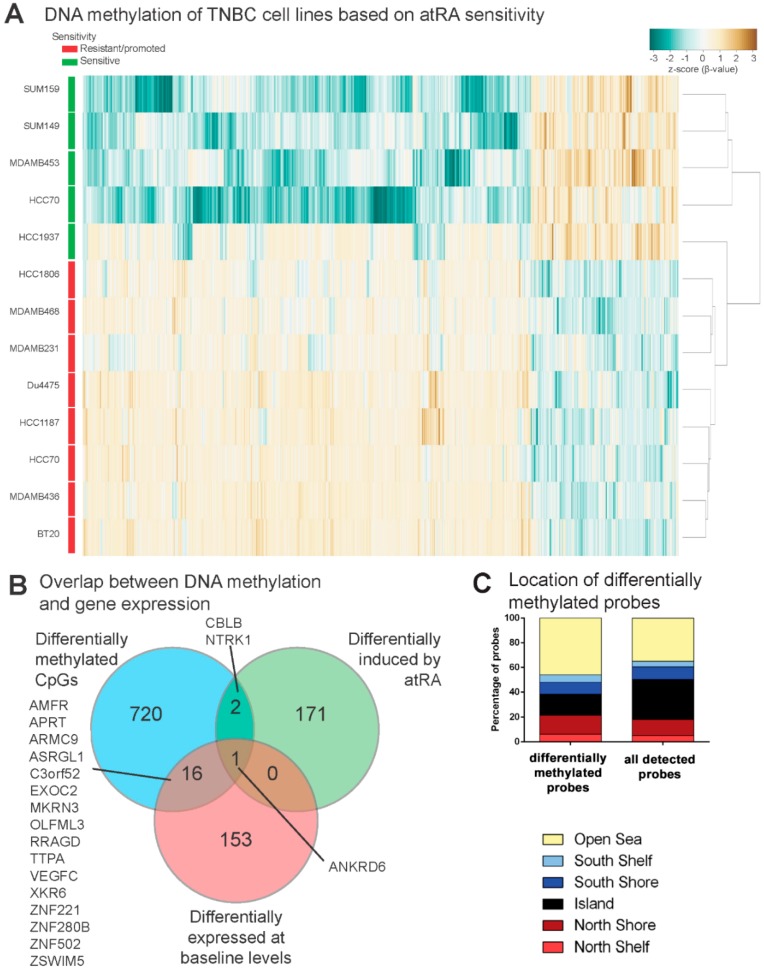
DNA methylation is a contributor to differences in baseline expression of identified genes. (**A**) Linear modelling of differences in CpG probe methylation between atRA-sensitive cell lines and all other cell lines identified 1409 sites which were significantly different. These were hierarchically clustered based on β-value. CpG identifiers were suppressed from the plot. (**B**) Gene lists were compared between differentially methylated probes, differentially expressed transcripts, and differences in atRA-inducibility between atRA-sensitive and all other cell lines. (**C**) Locations of differentially methylated probes (DMPs) relative to CpG islands are compared to all detected probes.

We have previously demonstrated that DNA methylation contributes to both baseline expression and the inducibility of atRA-responsive genes [16,33,41]. Therefore, we hypothesized that differences in DNA methylation may contribute to the atRA sensitivity of TNBC and could possibly be used to identify predictive atRA-inducible genes prior to treatment. We identified 1409 probes with differential methylation between atRA-sensitive and all other TNBC cell lines (Figure 4A, Supplementary File 4). We note with interest that although the response to atRA was greatest in HCC1937, it appears to be more similar to the non-responsive cell lines by hierarchical clustering. The 1409 probes were associated with 740 unique gene identifiers. We hypothesized that genes which have differentially methylated CpG dinucleotides in combination with differential expression are likely regulated by DNA methylation. The overlap between the genes with differentially methylated CpG probes, those identified as differentially expressed upon atRA treatment, and those which had differential basal expression was determined (Figure 4B), identifying 17 genes which we predicted would be regulated by DNA methylation. Using data available from The Cancer Genome Atlas (TCGA) [1], we correlated expression of four genes (MKRN3, TPPA, ZNF280B, and XKR6) with DNA methylation in 553 breast tumors (Supplementary Figure S2). We demonstrate strong correlations between methylation and expression for MKRN3, ZNF280B, and XKR6. Furthermore, we demonstrate that several of the CpG sites identified by this approach display varied patterns of DNA methylation within 1500 bp of the transcription start site (TSS) using the TNBC cell lines utilized in this work (Supplementary Figure S2). The HM450 array was designed with clear bias towards promoter-related CpG sites and genes of interest [45]. Given the inherent bias of using a gene-centric view of data from HM450 arrays (as in Figure 4C), we therefore did not exclude any distal probes from further analysis [46,47].

### 2.5. DNA Methylation Predicts Sensitivity of Four TNBC PDXs

Using the Affymetrix HuGene 2.0ST data for PDX A and B, we first characterized the intrinsic subtypes using the single-sample predictor and the claudin-low algorithm. PDX A and B were classified as basal-like, and PDX C and D have been previously identified as basal-like [48]. Baseline gene expression for all four PDXs was compared to the expression of TNBC cell lines (Supplementary Figure S3). Expression was row-centered for visualization. Hierarchical clustering reveals that all PDXs are most similar to atRA-resistant or -promoted cell lines. If baseline gene expression is predictive of response, we expected all PDXs will be resistant to atRA treatment in vivo.

**Figure 5:**
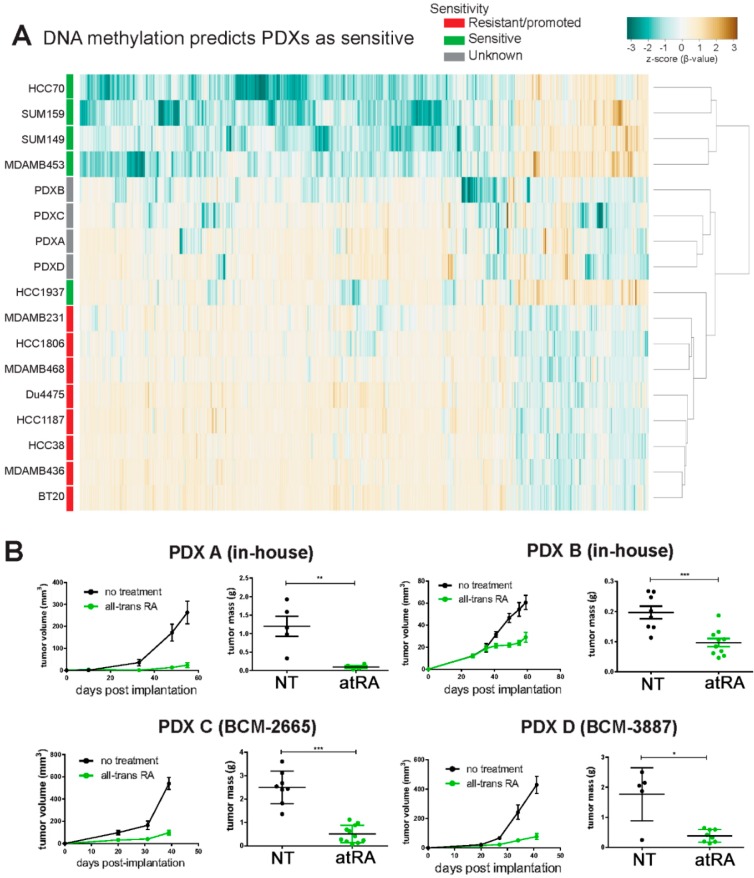
DNA methylation of 1409 probes predicts four patient-derived xenographs (PDXs) as atRA-sensitive. (**A**) β-values of CpG probes from analysis of atRA-sensitive cell lines and PDXs are compared by hierarchical clustering. PDXs cluster most closely with each other and are most similar to atRA-sensitive HCC1937. (**B**) Growth of PDXs A, B, C (BCM-2665), and D (BCM-3887) was quantified, and tumor weight determined at the conclusion of the experiment. Tumor weights were compared by student’s *t*-test, * *p* < 0.05, ** *p* < 0.01, *** *p* < 0.001.

We next investigated whether DNA methylation of tumor cells (sorted as demonstrated in Supplementary Figure S4) could provide a more robust model for predicting the sensitivity of PDXs. From the 1409 CpG sites we identified (Figure 4A), we selected those 1331 probes which appear on both the HM450 and EPIC arrays. We clustered the 13 TNBC cell lines with the four PDXs (Figure 5A). This approach demonstrates that all four PDX A, B, C, and D are most similar to themselves, illustrating a key technical challenge in comparing PDXs to in vitro cell lines. Regardless, they are more closely related to the atRA-sensitive cell lines, and cluster with the highly sensitive HCC1937 cell line. Therefore, if DNA methylation is predictive of in vivo response to atRA, then all four PDXs would be sensitive to atRA treatment in vivo.

Treatment of NOD-*scid* mice with slow release atRA pellets significantly decreased tumor volume and tumor weight of four PDXs (Figure 5B), which validated our DNA methylation profiling. This indicates successful preliminary identification of methylation biomarkers for atRA sensitivity. Given that neither methylation nor gene expression was a robust predictor of sensitivity, the 1331 CpG sites, which had been previously identified and are available on both the HM450 and EPIC arrays, were further refined to examine those probes that were significantly different (*p* < 0.01) between all sensitive cell lines and PDXs and all other cell lines. The top six probes were identified as associated with genes TBCD, CCDC112, KHDRBS2, CD97, DPF3, and LRP5 (Supplementary File 5).

### 2.6. Predicting Sensitive Patients from TCGA

**Figure 6:**
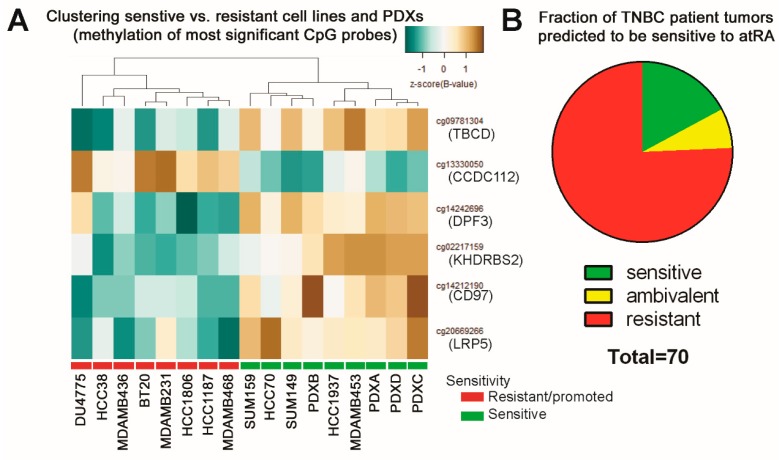
DNA methylation of six CpG sites predicts 17% of TNBCs as atRA-sensitive. (**A**) Hierarchical clustering of the six prioritized CpGs confirmed sensitivity of the four tested PDXs. The six probes were identified based on more stringent cutoffs (*p* < 0.01) between all sensitive cell lines and PDXs and all other cell lines. (**B**) Profiling and clustering of 70 TNBC samples from the TCGA Cell 2015 dataset was based on methylation of the six CpGs and indicated 17% as predicted-sensitive (Supplementary Figure S5).

Using TCGA data corresponding to TNBC patients [1], we extracted the HM450 β-values for the six prioritized CpG probes (Figure 6A). We clustered each of 70 samples individually with the 13 cell lines and four PDXs. Each sample was categorized as predicted-sensitive, ambiguous, or resistant (as in Supplementary Figure S5). We identified as many as 17% of samples that were predicted as sensitive (Figure 6B). This positions atRA as an underutilized clinical agent that could be of benefit to patients with TNBC.

## 3. Discussion

There is a growing need for novel biomarkers to predict how patients will respond to cancer therapies. Advances in genomic technologies are facilitating the identification of these biomarkers for a wide variety of drugs and improving the application of precision medicine. Several examples include the v600E BRAF mutation, which predicts response to specific BRAF inhibitors [49], as well as high BRCA1 expression predicting resistance to neoadjuvant gemcitabine/cisplatin chemotherapy in lung cancer [50]. However, predictive biomarkers do not guarantee successful therapy. For example, while KRAS mutations predict that EGFR inhibitors are not beneficial, not all individuals with wildtype KRAS exhibit measurable responses to cetuximab [51]. The marked genomic advances also allow for the reconsideration of drugs which have previously shown limited benefit in clinical settings. We hypothesized that improved patient stratification could amplify the clinical benefit of these existing therapies and investigated a potential stratification approach to predict response to atRA.

Despite their promise as differentiating agents, retinoids have seen limited clinical success in solid tumors. The establishment of a signature describing sensitivity to atRA would thus be a useful tool in accelerating future clinical studies. While some efforts have been made to describe atRA sensitivity in breast cancer models, these predominantly in vitro studies have designated most TNBC cells as resistant. In contrast, using TNBC cell-line xenografts, we describe a high degree of variability in atRA sensitivity. The use of TNBC models exclusively allowed for the elimination of much variability originating from inter-subtype heterogeneity. To our knowledge, this is the first time a panel of TNBC cell line and patient-derived xenografts have been characterized in vivo for atRA sensitivity. We utilized gene expression and DNA methylation to predict atRA sensitivity of four basal-like PDXs and validated DNA methylation as a stratification approach for these PDXs. This is an important description of an atRA-sensitive profile which can be used for further investigation of atRA in clinical studies.

Biomarker discovery has mostly centered around mutations and gene expression, with other omics such as methylome and proteome are not yet capitalized on. This is an area of enormous potential. DNA is more stable than RNA, increasing the likelihood that it can be extracted at high quality from tissues or liquid biopsies. Specifically, DNA methylation as a biomarker could be translated to circulating tumor DNA assays, potentially allowing non-invasive predictions of therapeutic response. Methylation is already used as a predictive biomarker, for instance, methylation of O6-methylguanine-DNA methyltransferase gene (MGMT) in glioma predicts better response to alkylating agents [52]. However, it is not in wide use due to numerous technical challenges which limit broad applicability [53,54]. While we did identify a consistent methylation profile between atRA-sensitive cell lines and PDXs, it is also likely that DNA methylation will vary depending on the growth environment: in vitro, in vivo, ex vivo, or in the natural host [55,56]. Additionally, non CpG-methylation, while not considered in this study, is an emerging area of gene regulation in specific cell types, including stem cells and could also be useful for biomarker consideration [57,58]. The correlation between non-CpG methylation and gene expression has not been fully characterized, particularly in the complex and dynamic environment of a tumor [59,60]. The biomarkers identified in this study are purely correlative, and their functional relevance in the effects of atRA on cell proliferation or differentiation have not yet been investigated. Given the complex network governed by retinoid signaling [33], there is a high probability that these biomarkers may be explainable as distal regulatory elements. Future studies may identify possible combination therapies, such as retinoids with chromatin modifying drugs, which enhance the potency of retinoid therapy [61]. Additionally, further investigations using genomic sequencing of the TNBC cell lines utilized in this study may identify additional, relevant mutations which may correlate with atRA sensitivity and may be associated with distal regulatory elements [62].

Considering that cell-line xenografts may have limited clinical relevance and that the in vitro biomarker discovery may not be entirely transferable to in vivo usage, we utilized four PDXs in this study and demonstrate potential benefit of atRA treatment. However, there is limited heterogeneity in the PDXs used (all four were basal-like TNBC). Although basal-like tumors represent the majority of TNBCs [9], the inclusion of other subtypes would give a broader applicability to potential clinical use of atRA. Further pre-clinical investigations which expand the modeling of atRA sensitivity and resistance in PDXs will improve the robustness of this predictive profile. This would provide an opportunity to develop detection of methylation biomarkers in circulating tumor (ct) DNA and could accelerate the clinical introduction of predictive identification of atRA sensitivity.

## 4. Materials and Methods

### 4.1. Cell Culture and Reagents

With the exception of SUM149 and SUM159 cells that were obtained from BioIVT (previously Asterand, Detroit, MI, USA), all other cell lines were obtained from the American Type Culture Collection (ATCC) and maintained as recommended by supplier. Where indicated, all-trans retinoic acid (atRA, Sigma-Aldrich, Oakville, ON, Canada) was used at 100 nM for 18 h.

### 4.2. Cell-Line Xenografts

One day prior to tumor-cell implantation, experimental mice were implanted with a slow-release atRA pellet (5 mg/60 days, Innovative Research of America, Sarasota, FL, USA). Cells were admixed 1:1 with high concentration Matrigel (Corning, New York, NY, USA)and injected orthotopically into the mammary fat pad of female NOD-*scid* mice (HCC38, HCC70, MDA-MB-436, MDA-MB-453, HCC1187, BT20, HCC1937, Du4475: 5 × 10^6^ cells/mouse; MDA-MB-231, MDA-MB-468, SUM159, SUM149: 2 × 10^6^ cells/mouse; and HCC1806: 1 × 10^5^ cells/mouse). Primary tumor volume was quantified (mm^3^, length × width × depth/2) for the duration of the experiment. Tumor-bearing mice were euthanized and tumor weight quantified.

### 4.3. Patient-Derived Xenografts

PDX A and B were derived from patients at the QEII Health Sciences Center (QEII HSC, Halifax, NS, Canada). Surgical biopsies from primary tumors were harvested and stored in DMEM with 10% FBS for <1 h until implantation. 2–3 mm^3^ pieces were sutured to the thoracic mammary fat pad of female NOD-*scid* mice. After palpable tumors developed, mice were euthanized, tumors harvested, and 2–3 mm^3^ pieces were successively implanted.

PDX C (BCM-3887) and PDX D (BCM-2665) were obtained from the laboratory of Dr. Michael Lewis (Baylor College of Medicine, Houston, TX, USA) as frozen samples [48]. Upon receipt, they were surgically implanted into the mammary fat pad of female NOD-*scid* mice for expansion, and subsequently preserved in liquid nitrogen.

### 4.4. Q Relative Real-Time PCR

Total RNA was extracted using Trizol reagent and the PureLink RNA kit (Thermo Fisher Scientific, Mississauga, ON, Canada) with DNase treatment. Equal amounts of RNA were reverse-transcribed using iScript (Bio-Rad, Mississauga, ON, Canada), and relative real-time PCR (qPCR) was performed using gene-specific primers (Supplementary Table S2). Standard curves for each primer set were generated, and primer efficiencies were incorporated into the CFX Manager software (Bio-Rad). mRNA expression of all samples was calculated relative to two reference genes, glyceraldehyde 3-phosphate dehydrogenase (GAPDH) and β-2-microglobulin (B2M).

### 4.5. Preparation of Cells from PDXs

PDX-bearing mice were euthanized, and tumors harvested. Tumors were minced and incubated in collagenase (225 U/mL; BioShop Canada Inc., Burlington, ON, Canada) in HBSS, Thermo Fisher Scientific, Mississauga, ON, Canada) at 37 °C on an end-over-end shaker. After 2 h, cell suspension was passed through a 70 µm strainer (Fisher Scientific) and centrifuged for 5 min at 500× *g*. Cells were resuspended in red blood cell lysis buffer (150 mM NH_4_Cl, 10 mM KHCO_3_, 0.1 mM Na_2_EDTA). After 5 min, cells were centrifuged, resuspended in PBS, and passed through a 70 µm strainer. Cells were centrifuged, resuspended in Aldefluor buffer (Stem Cell Technologies, Inc., Vancouver, BC, Canada), and passed through a 70 µm strainer. Approximately 1 × 10^7^ cells were incubated with anti-H-2Kd (1:1000 SF1-1.1, BioLegend, San Diego, CA, USA) at 37 °C with shaking. After 1 h, cells were centrifuged and resuspended in Aldefluor buffer with 7-AAD (1:10, Biolegend). Stained cells were gated on SSC and FSC to eliminate doublets. 7-AAD^-^ H-2Kd^-^ cells were sorted into ice-cold PBS with 5% BSA (Sigma-Aldrich). H-2Kd purity was assessed (FACS Aria, BD Bioscience, San Jose, CA, USA).

### 4.6. Preparation of Cell Line Samples for Arrays and Analysis

TNBC cells were treated with atRA; total RNA was extracted as described. Sample preparation, amplification, hybridization to the Affymetrix HuGene 2.0 ST array, and data collection were performed by The Centre for Applied Genomics at the Hospital for Sick Children (Toronto, ON, Canada) and data is accessible from GSE117579. HuGene 2.0 ST data for MDA-MB-231 and MDA-MB-468 cells with and without atRA treatment was obtained from GSE103426 [33]. Expression data was analyzed in the R environment using the oligo package with RMA normalization [63].

HM450 data for all cell lines was obtained from GSE78875 [41], and analyzed in the R environment using the minfi package with Swan normalization [64,65]. Cross-reactive and SNP-associated probes were removed.

### 4.7. Preparation of Patient-Derived Xenografts for Arrays and Analysis

DNA was extracted from human cells of PDXs using the PureLink DNA kit (Thermo Fisher Scientific), according to manufacturer’s instructions. Sample preparation, bisulfite conversion, hybridization to the Illumina EPIC array (PDX C and D), and data collection were performed by The Centre for Applied Genomics (TCAG) at the Hospital for Sick Children (Toronto, ON, Canada). Data can be accessed through the GEO repository (GSE117926). HM450 data (PDX A and B) was obtained from GSE78875 [41]. HM450 and EPIC data was analyzed in the R environment using the minfi package with Swan normalization [64,65]. Cross-reactive and SNP-associated probes were removed.

RNA was extracted from all cells of PDXs using Trizol reagent and the PureLink RNA kit (Invitrogen) with DNase treatment. Sample preparation, labelling, and hybridization to the Affymetrix HuGene 2.0ST array was performed by TCAG and data can be accessed through the GEO repository (PDX A and B, GSE117579; PDX C and D, GSE118002).

### 4.8. Subtyping of Patient-Derived Xenografts

PDXs were subtyped with normalized RNA expression using SSP2003 via the genefu package in the R environment [66,67]. The claudin-low classification algorithm was also applied in genefu [5].

### 4.9. cBioPortal Analyses

Data from TCGA [1] was extracted for expression correlations using cBioPortal [68,69]. Only those 533 patients with complete RNAseq and HM450 data were utilized. Spearman’s correlations were calculated between RNAseq and HM450 methylation. Data was extracted in the R environment using the TCGAbiolinks package for patient stratification [70].

### 4.10. Statistical Analyses

All statistical analyses beyond those described in the R environment were conducted with GraphPad Prism 6.0. Tumor weights were compared by student’s *t*-test. The molecular subtype and the presence of alterations in driver genes were compared between sensitive cell lines and all remaining cell lines by a Fisher’s exact test. For all analyses, * *p* < 0.05, ** *p* <0.01, *** *p* < 0.001.

### 4.11. Study Approval

All experiments were conducted in accordance with the Canadian Council on Animal Care standards and protocols approved by Dalhousie University Committee on Laboratory Animals (#15-013 and #17-011). PDX A and B patient samples were collected, maintained, and analyzed in accordance with protocol #1007106, approved by the IWK Health Centre Research Ethics Board.

## 5. Conclusions

We detail a methylation-based predictive profile to identify atRA sensitivity in TNBC. Importantly, if proven successful for atRA, it may be possible to use a similar differential DNA methylation profiling strategy to predict sensitivity to other drugs, including more potent and selective retinoids and rexinoids. Hence, these findings provide a paradigm for utilizing DNA methylation as a stratification approach for treatment selection.

## Supplementary Materials

The following are available online at http://www.mdpi.com/2072-6694/10/11/397/s1, Figure S1: Methylation of RARB2 does not correlate with atRA sensitivity; Figure S2: Patterns of methylation differ between atRA-sensitive cell lines and remaining TNBC cell lines; Figure S3: Baseline gene expression does not accurately predict response of TNBC PDXs to atRA; Figure S4: Live human cells are isolated from PDXs; Figure S5: Hierarchical clustering identifies 12 TNBC patients as potentially sensitive to atRA treatment; Table S1: Literature summary details varied responses of TNBC cell lines to retinoids; Table S2: Primers utilized for qPCR; File S1: Differential baseline expression; File S2: Differential atRA expression; File S3: Induced expression in 13 cell lines; File S4: DMPs; File S5: DMPs including PDX.
