# Supplementary material for: DNA Methylation Predicts the Response of Triple-Negative Breast Cancers to All-Trans Retinoic Acid
Source: Cancers (Basel). 2018 Oct 24;10(11):397. doi: 10.3390/cancers10110397 (PMC6266978; doi:10.3390/cancers10110397)
Supplement: Supplementary file 1 [file cancers-10-00397-s001.zip › cancers-368551-Supplementary Figures and Tables-final-.docx]

*Supplementary material*

DNA Methylation Predicts the Response of Triple-Negative Breast Cancers to All-Trans Retinoic Acid

Krysta Mila Coyle, Cheryl A. Dean, Margaret Lois Thomas, Dejan Vidovic, Carman A. Giacomantonio, Lucy Helyer and Paola Marcato


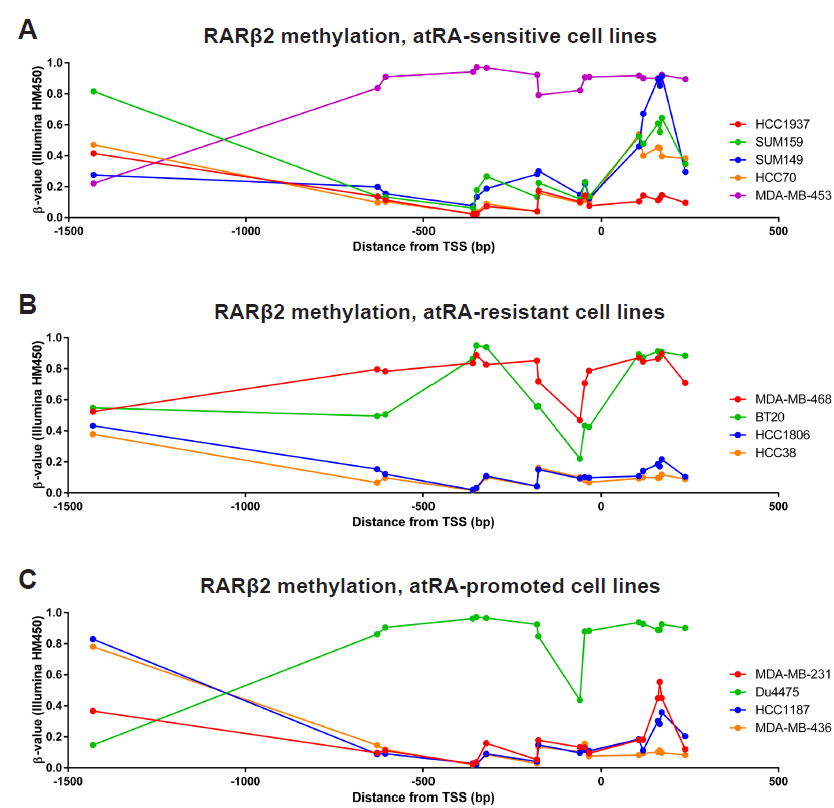


**Figure S1.** Methylation of RARB2 does not correlate with atRA sensitivity. B-values for the region extending 1500 bp in either direction from the transcription start site (TSS) of RARB2 were extracted for all indicated cell lines from GSE78875 [1]. Values are plotted for (**A**) atRA sensitive cell lines; (**B**) atRA resistant cell lines; and (**C**) atRA promoted cell lines.


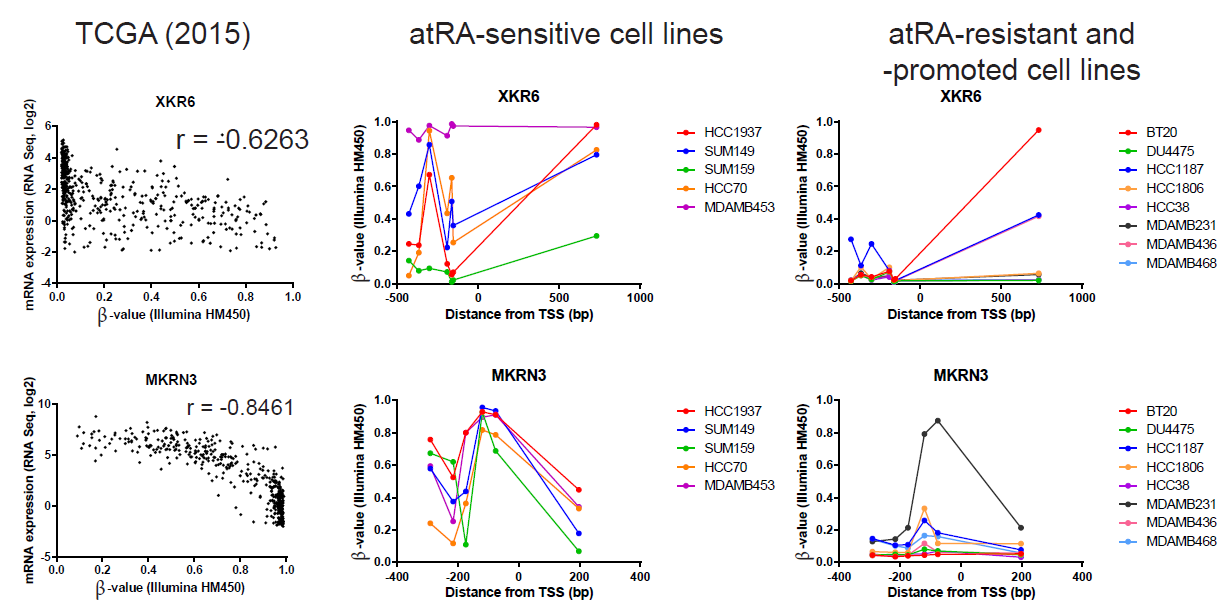

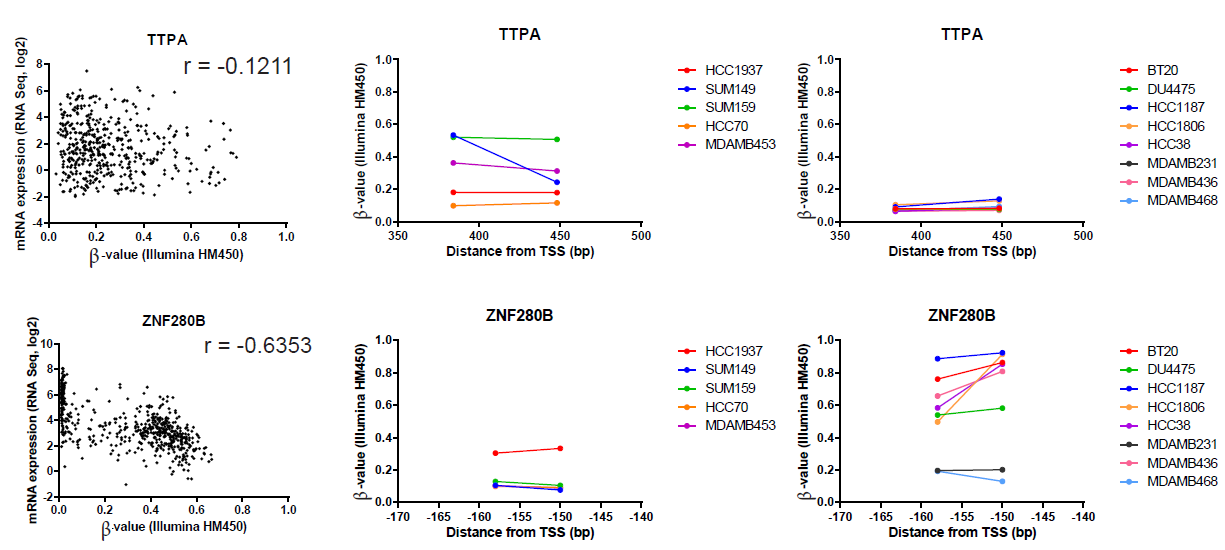


**Figure S2.** Patterns of methylation differ between atRA-sensitive cell lines and remaining TNBC cell lines. Based on the differentially methylated probes identified, data from TCGA was extracted to identify correlations between methylation and gene expression. B-values for the selected probes within 1500 bp of the TSS of indicated genes were extracted from HM450 data for all cell lines as shown. atRA sensitive cell lines are plotted independently of all other cell lines.


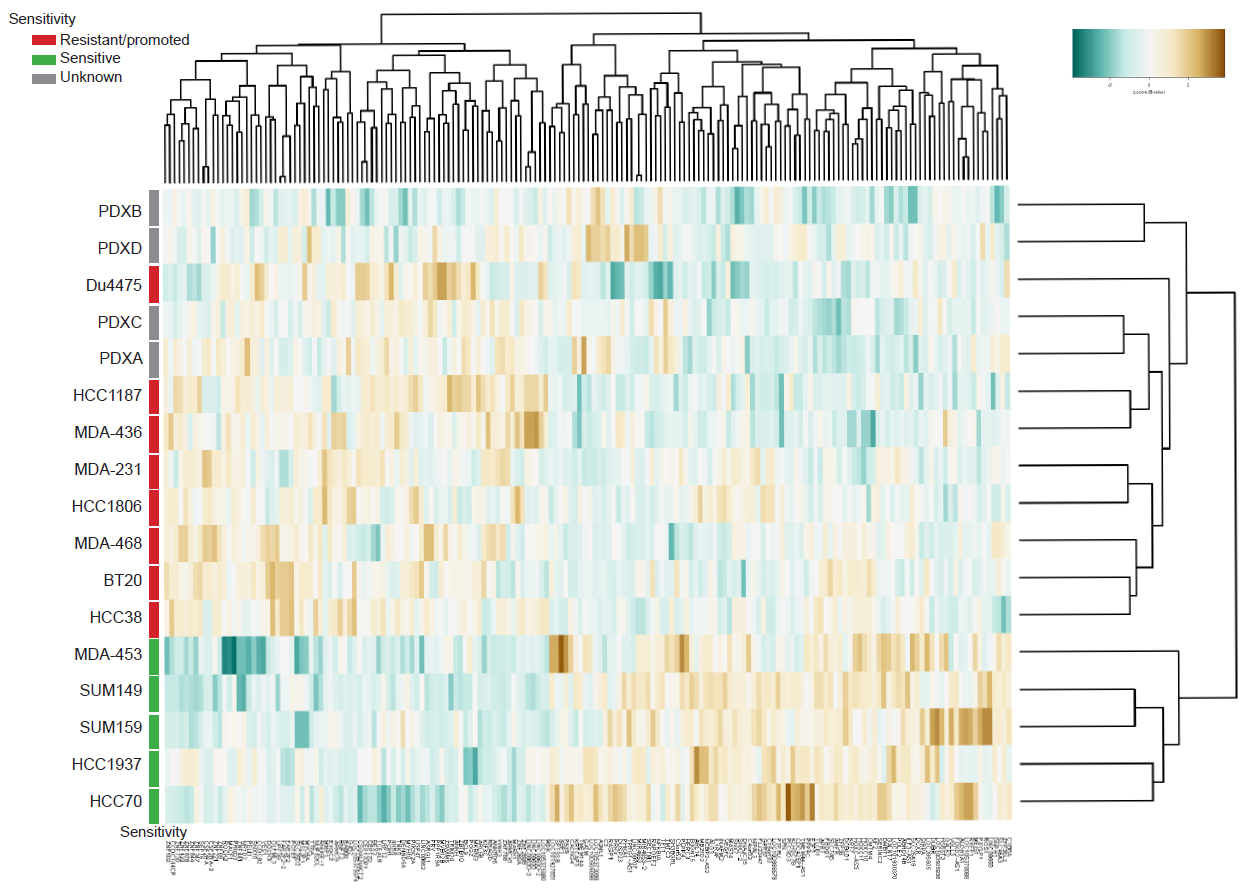


**Figure S3.** Baseline gene expression does not accurately predict response of TNBC PDXs to atRA. Utilizing the genes identified from the cell-line panel (sensitive vs. other), PDXs A–D were added. Hierarchical clustering predicts PDXs as resistant to atRA.


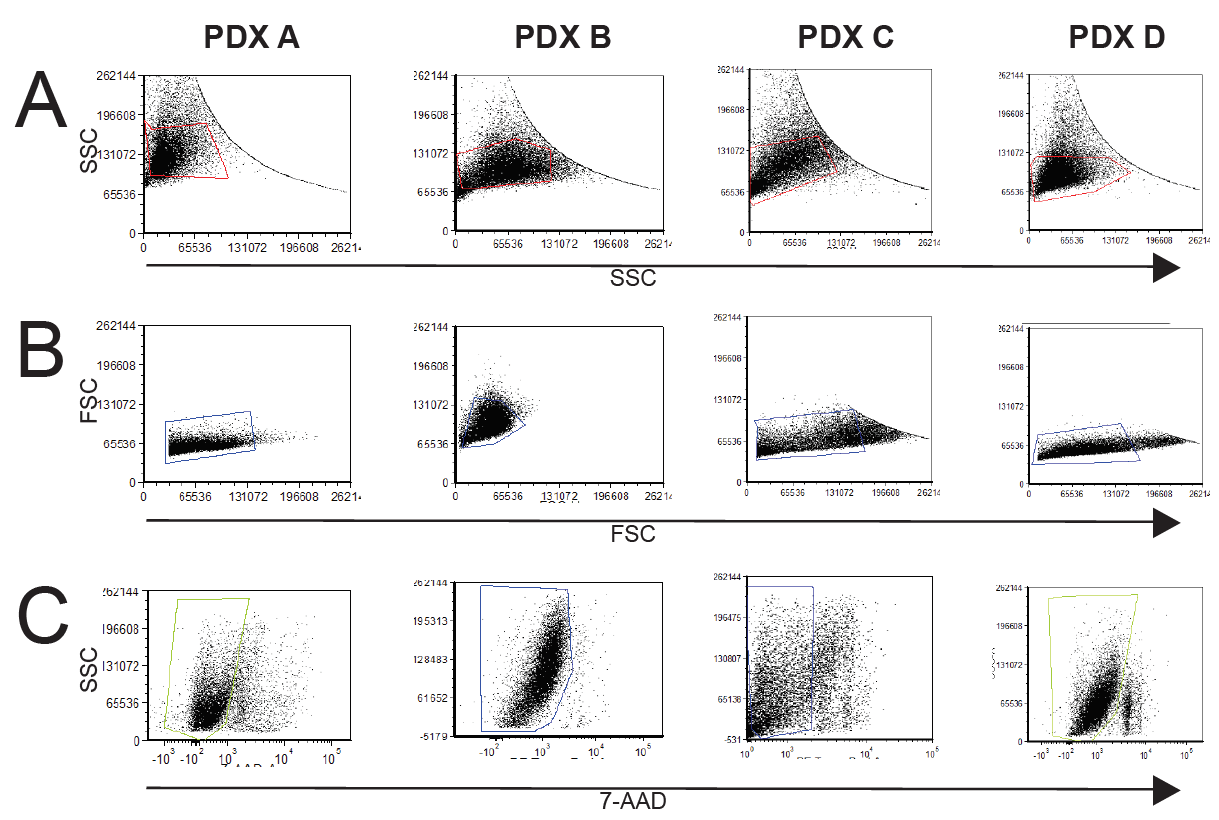

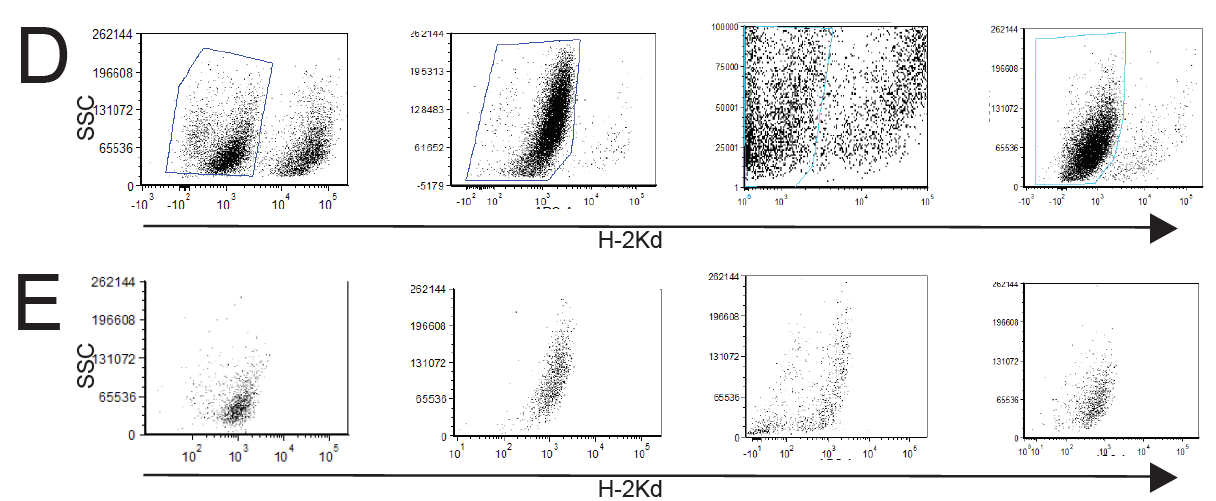


**Figure S4.** Live human cells are isolated from PDXs. Prepared and stained cells from PDXs A–D were gated and sorted to **A**. exclude doublets based on side scatter (SSC); **B**. exclude doublets based on forward scatter (FSC); **C**. identify 7-AAD- live cells; and **D**. identify H-2Kd- human cells. **E**. H-2Kd purity of sorted cells was verified.


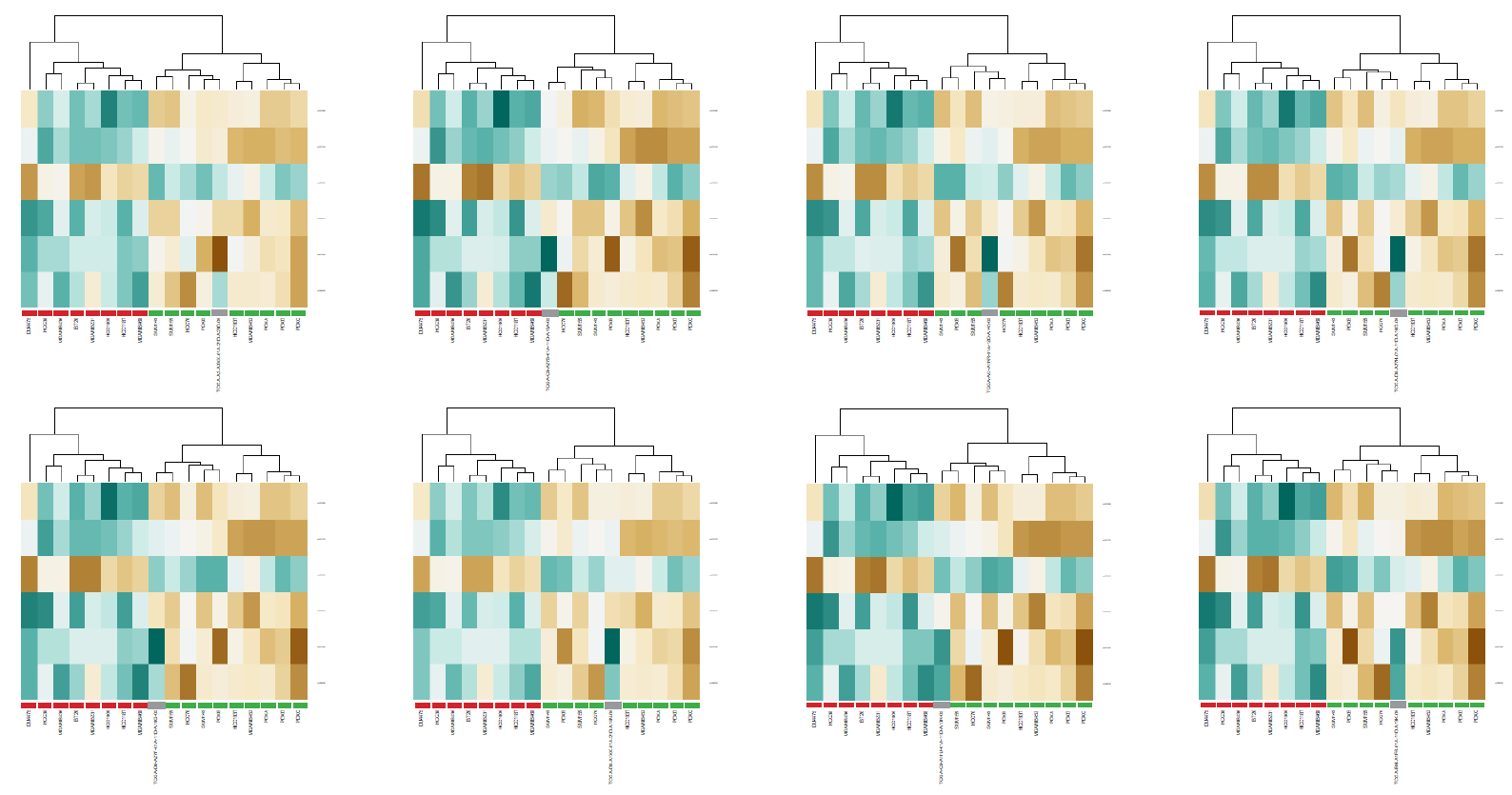


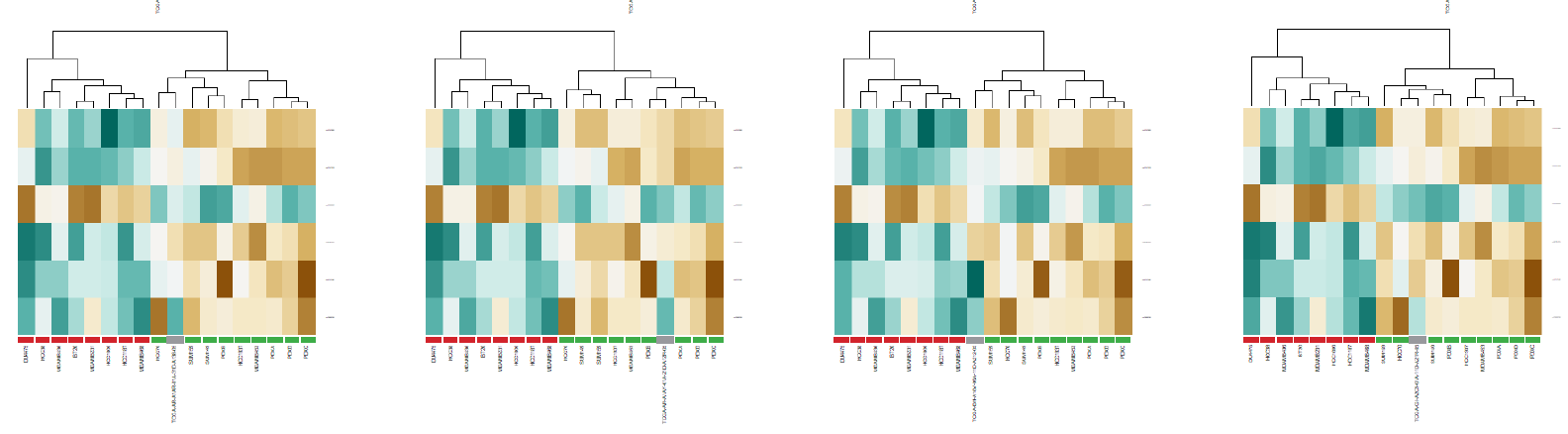

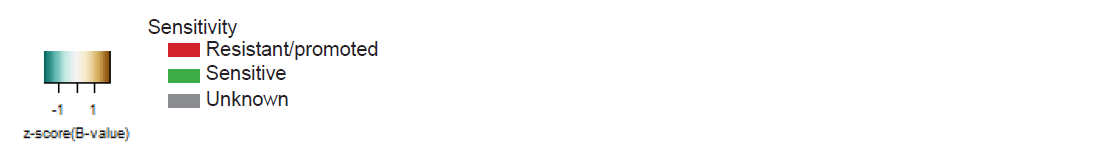


**Figure S5.** Hierarchical clustering identifies 12 TNBC patients as potentially sensitive to atRA treatment. B-values for 6 CpGs were extracted for TNBC patients with HM450 genomic methylation data. Individual patients were hierarchically clustered with 13 profiled TNBC cell lines and 4 profiled PDXs. Clustering with sensitive cell lines and PDXs indicates hypothetical sensitivity.

**Table S1.** Literature summary details varied responses of TNBC cell lines to retinoids.

| **TNBC Cell Line** | **Evaluation** | **Model** | **References** |
| --- | --- | --- | --- |
| HCC1937 | **Resistant** | In vitro | [2,3] |
| SUM159 | **Resistant** | In vitro | [4] |
|  | Sensitive | Tumorsphere | [5] |
|  |  | In vivo (intermediate) | [4] |
| SUM149 | **Resistant** | Tumorsphere | [6] |
|  |  | In vitro | [4] |
|  | Sensitive | Tumorsphere | [5] |
| HCC70 | Sensitive | In vitro (intermediate) | [2] |
| MDA-MB-453 | **Resistant** | In vitro | [2,7,8] |
| MDA-MB-468 | **Resistant** | In vitro | [2,9] |
|  |  | In vivo | [10] |
|  | Sensitive | In vivo | [3] |
| BT20 | **Resistant** | In vitro | [2,9] |
|  |  | Tumorsphere | [6] |
| HCC1806 | Sensitive | In vitro (intermediate) | [2,11] |
| HCC38 | **Resistant** | In vitro | [2] |
| MDA-MB-231 | **Resistant** | In vitro | [2,4,7,9,11,12] |
|  |  | Tumorsphere | [6] |
|  |  | In vivo | [4] |
|  | **Promoted** | In vivo | [10] |
|  | **Resistant** (***13-cis RA***) | In vivo | [12] |
|  | Sensitive | In vivo | [3] |
|  |  | In vitro (intermediate) |  |
|  | Sensitive (retinol, retinyl palmitate) | In vivo | [13,14] |
| Du4475 | **Resistant** | In vitro | [2] |
| HCC1187 | *No evidence* |  |  |
| MDA-MB-436 | **Resistant** | In vitro | [2] |

N.B. ‘Intermediate’ refers to a classification of intermediate sensitivity.

**Table S2.** Primers utilized for qPCR.

| **Gene Symbol** | **Primer Sequence (5'–3')** | | **Reference** |
| --- | --- | --- | --- |
| ALDH1A1 | F | TGTTAGCTGATGCCGACTTG | [15] |
|  | R | TTCTTAGCCCGCTCAACACT |  |
| ALDH1A2 | F | CTGGCAATAGTTCGGCTCTC | [15] |
|  | R | TGATCCTGCAAACACTGCTC |  |
| ALDH1A3 | F | TCTCGACAAAGCCCTGAAGT | [15] |
|  | R | TATTCGGCCAAAGCGTATTC |  |
| ALDH8A1 | F | TGGTGAGCATAGGTGCTCTG | [15] |
|  | R | GTTATCACCGTGGGAAGCAT |  |
| CRABP2 | F | ACTGACCAACGATGGGGAAC |  |
|  | R | ACTCTCGGACGTAGACCCTG |  |
| CRBP1 | F | GTCGACTTCACTGGGTACTGG |  |
|  | R | GCTTCAGCAAGTTGGCGATT |  |
| CYP26A1 | F | TGTTGATCGAGCACTCGTGG |  |
|  | R | TCAGAGATGTGGCTGCACTG |  |
| CYP26B1 | F | CATGGGCTTCCCGCTCAT |  |
|  | R | TGGATCTTGGGCAGGTAACTC |  |
| CYP26C1 | F | GTTCCCTTCAGTGGCCTACG |  |
|  | R | CTTCTCAGAAATGGCCCCCTC |  |
| EP300 | F | ATCCAGGGCCTAACATGGGA |  |
|  | R | AGGCATCATCTGGTTTGGCA |  |
| FABP5 | F | CACAGCTGATGGCAGAAAAACT |  |
|  | R | CTTCCCATCCCACTCCTGATG |  |
| NCOR2 | F | CTGAAGCCACTGTCAACAACAG |  |
|  | R | ATTCTGCCCTGTGTCCTTGG |  |
| RARα | F | CGGGTGATCACGCTGAAGAT |  |
|  | R | GGCCCTCTGAGTTCTCCAAC |  |
| RARβ | F | GGTTTCACTGGCTTGACCAT | [10] |
|  | R | GGCAAAGGTGAACACAAGGT |  |
| RARγ | F | CTGGAGATGGATGACACCGAG |  |
|  | R | GCTTGTCCACTTTTTCGGGC |  |
| RXRα | F | CCAAGACCGAGACCTACGTG |  |
|  | R | CCACTCCACCAGGGTGAAAA |  |
| RXRβ | F | TCCTCCTTGCCACAGGTCTT |  |
|  | R | GAGGGACCGATCAAAGATGGC |  |
| RXRγ | F | GTTGTGAAGGCTGCAAAGGG |  |
|  | R | CGCTGACGCTTGTCAATGAG |  |
| GAPDH | F | GGAGTCAACGGATTTGGTCGTA | [10] |
|  | R | TTCTCCATGGTGGTGAAGAC |  |
| B2M | F | AGGCTATCCAGCGTACTCCA | [1] |
|  | R | CGGATGGATGAAACCCAGACA |  |

References

1. Coyle, K.M.; Murphy, J.P.; Vidovic, D.; Vaghar-Kashani, A.; Dean, C.A.; Sultan, M.; Clements, D.; Wallace, M.; Thomas, M.L.; Hundert, A.; et al. Breast cancer subtype dictates DNA methylation and ALDH1A3-mediated expression of tumor suppressor RARRES1. *Oncotarget* **2016**, doi:10.18632/oncotarget.9858.
2. Centritto, F.; Paroni, G.; Bolis, M.; Garattini, S. K.; Kurosaki, M.; Barzago, M. M.; Zanetti, A.; Fisher, J.N.; Scott, M.F.; Pattini, L.; et al. Cellular and molecular determinants of all-trans retinoic acid sensitivity in breast cancer: Luminal phenotype and RARα expression. *EMBO Mol. Med.* **2015**, *7*, 950–972, doi:10.15252/emmm.201404670.
3. Wei, S.; Kozono, S.; Kats, L.; Nechama, M.; Li, W.; Guarnerio, J.; Luo, M.; You, M.-H.; Yao, Y.; Kondo, A.; et al. Active Pin1 is a key target of all-trans retinoic acid in acute promyelocytic leukemia and breast cancer. *Nat. Med.* **2015**, *21*, 457–466, doi:10.1038/nm.3839.
4. Merino, V.F.; Nguyen, N.; Jin, K.; Sadik, H.; Cho, S.; Korangath, P.; Han, L.; Foster, Y.M.N.; Zhou, X.C.; Zhang, Z.; et al. Combined treatment with epigenetic, differentiating, and chemotherapeutic agents cooperatively targets tumor-initiating cells in triple-negative breast cancer. *Cancer Res.* **2016**, *76*, 2013–2024, doi:10.1158/0008-5472.CAN-15-1619.
5. Ginestier, C.; Wicinski, J.; Cervera, N.; Monville, F.; Finetti, P.; Bertucci, F.; Wicha, M.S.; Birnbaum, D.; Charafe-Jauffret, E. Retinoid signaling regulates breast cancer stem cell differentiation. *Cell Cycle* **2009**, *8*, 3297–3302.
6. Wu, M.-J.; Kim, M.R.; Chen, Y.-S.; Yang, J.-Y.; Chang, C.-J. Retinoic acid directs breast cancer cell state changes through regulation of TET2-PKCζ pathway. *Oncogene* **2017**, *36*, 3193, doi:10.1038/onc.2016.467.
7. Takatsuka, J.; Takahashi, N.; de Luca, L.M. Retinoic acid metabolism and inhibition of cell proliferation: an unexpected liaison. *Cancer Res.* **1996**, *56*, 675–678.
8. Tari, A.M.; Lim, S.-J.; Hung, M.-C.; Esteva, F.J.; Lopez-Berestein, G. Her2/neu induces all-trans retinoic acid (ATRA) resistance in breast cancer cells. *Oncogene* **2002**, *21*, 5224–5232, doi:10.1038/sj.onc.1205660.
9. Liu, Y.; Lee, M.O.; Wang, H.G.; Li, Y.; Hashimoto, Y.; Klaus, M.; Reed, J.C.; Zhang, X. Retinoic acid receptor beta mediates the growth-inhibitory effect of retinoic acid by promoting apoptosis in human breast cancer cells. *Mol. Cell. Biol.* **1996**, *16*, 1138–1149, doi:10.1128/MCB.16.3.1138.
10. Marcato, P.; Dean, C.A.; Liu, R.-Z.; Coyle, K.M.; Bydoun, M.; Wallace, M.; Clements, D.; Turner, C.; Mathenge, E.G.; Gujar, S.A.; et al. Aldehyde dehydrogenase 1A3 influences breast cancer progression via differential retinoic acid signaling. *Mol. Oncol.* **2015**, *9*, 17–31, doi:10.1016/j.molonc.2014.07.010.
11. Lin, G.; Zhu, S.; Wu, Y.; Song, C.; Wang, W.; Zhang, Y.; Chen, Y.-L.; He, Z. ω-3 free fatty acids and all-trans retinoic acid synergistically induce growth inhibition of three subtypes of breast cancer cell lines. *Sci. Rep.* **2017**, *7*, doi: 10.1038/s41598-017-03231-9.
12. Wu, Q.; Dawson, M.I.; Zheng, Y.; Hobbs, P.D.; Agadir, A.; Jong, L.; Li, Y.; Liu, R.; Lin, B.; Zhang, X.K. Inhibition of trans-retinoic acid-resistant human breast cancer cell growth by retinoid X receptor-selective retinoids. *Mol. Cell. Biol.* **1997**, *17*, 6598–6608.
13. Halter, S.; Fraker, L.; Adcock, D.; Vick, S. Effect of retinoids on xenotransplanted human mammary carcinoma cells in athymic micee. *Cancer Res.* **1988**, *48*, 3733–3736.
14. Fraker, L.; Halter, S.; Forbes, J. Growth inhibition by retinol of a human breast carcinoma cell line in vitro and in athymic mice. *Cancer Res.* **1984**, *44*, 5757–5763.
15. Marcato, P.; Dean, C.A.; Da, P.; Araslanova, R.; Gillis, M.; Joshi, M.; Helyer, L.; Pan, L.; Leidal, A.; Gujar, S.; et al. Aldehyde dehydrogenase activity of breast cancer stem cells is primarily due to isoform ALDH1A3 and its expression is predictive of metastasis. *Stem Cells* **2011**, *29*, 32–45.

© 2018 by the authors. Submitted for possible open access publication under the terms and conditions of the Creative Commons Attribution (CC BY) license (http://creativecommons.org/licenses/by/4.0/).
